# Modelling the impact of CD4 testing on mortality from TB and cryptococcal meningitis among patients with advanced HIV disease in nine countries

**DOI:** 10.1002/jia2.26070

**Published:** 2023-03-07

**Authors:** Ikwo Kitefre Oboho, Heather Paulin, Carl Corcoran, Matt Hamilton, Alex Jordan, Hannah L. Kirking, Elfriede Agyemang, Laura Jean Podewils, Carel Pretorius, Greg Greene, Tom Chiller, Mitesh Desai, Roma Bhatkoti, Ray W. Shiraishi, N. Sarita Shah

**Affiliations:** ^1^ Division of Global HIV and TB Centers for Disease Control and Prevention Atlanta Georgia USA; ^2^ Avenir Health Glastonbury Connecticut USA; ^3^ Division of Foodborne Waterborne and Environmental Diseases Centers for Disease Control and Prevention Atlanta Georgia USA; ^4^ Denver Health and Hospital Authority Denver Colorado USA; ^5^ U.S. Office of Global AIDS Coordinator and Health Diplomacy Washington DC USA; ^6^ Emory Rollins School of Public Health Atlanta Georgia USA

**Keywords:** advanced HIV disease, CD4 testing, cryptococcal meningitis, deaths averted, mortality, TB mortality

## Abstract

**Introduction:**

Despite antiretroviral therapy (ART) scale‐up among people living with HIV (PLHIV), those with advanced HIV disease (AHD) (defined in adults as CD4 count <200 cells/mm^3^ or clinical stage 3 or 4), remain at high risk of death from opportunistic infections. The shift from routine baseline CD4 testing towards viral load testing in conjunction with “Test and Treat” has limited AHD identification.

**Methods:**

We used official estimates and existing epidemiological data to project deaths from tuberculosis (TB) and cryptococcal meningitis (CM) among PLHIV‐initiating ART with CD4 <200 cells/mm^3^, in the absence of select World Health Organization recommended diagnostic or therapeutic protocols for patients with AHD. We modelled the reduction in deaths, based on the performance of screening/diagnostic testing and the coverage and efficacy of treatment/preventive therapies for TB and CM. We compared projected TB and CM deaths in the first year of ART from 2019 to 2024, with and without CD4 testing. The analysis was performed for nine countries: South Africa, Kenya, Lesotho, Mozambique, Nigeria, Uganda, Zambia, Zimbabwe and the Democratic Republic of Congo.

**Results:**

The effect of CD4 testing comes through increased identification of AHD and consequent eligibility for protocols for AHD prevention, diagnosis and management; algorithms for CD4 testing avert between 31% and 38% of deaths from TB and CM in the first year of ART. The number of CD4 tests required per death averted varies widely by country from approximately 101 for South Africa to 917 for Kenya.

**Conclusions:**

This analysis supports retaining baseline CD4 testing to avert deaths from TB and CM, the two most deadly opportunistic infections among patients with AHD. However, national programmes will need to weigh the cost of increasing CD4 access against other HIV‐related priorities and allocate resources accordingly.

## 1 INTRODUCTION

The scale‐up of antiretroviral therapy (ART) over the past decade has reduced morbidity and mortality among people living with HIV (PLHIV) [1]. However, mortality remains highest among those with advanced HIV disease (AHD, in adults defined as CD4 count <200 cells/mm^3^ or World Health Organization [WHO] clinical stage 3 or 4 disease), at an increased risk for opportunistic infections (OIs), including tuberculosis (TB) and cryptococcal meningitis (CM) [2, 3]. TB is the leading cause of morbidity and mortality among PLHIV, accounting for one‐third of all deaths [4]. CM accounts for 15–20% of deaths among hospitalized adult PLHIV and is the most common cause of meningitis in adult PLHIV in sub‐Saharan Africa (SSA) [5, 6, 7]. While CD4 count at ART initiation has significantly improved with “Test and Treat,” whereby PLHIV initiate ART irrespective of CD4, up to one‐third of PLHIV still initiate ART with AHD [8, 9, 10].

The WHO 2017 guidelines promote a package of care for persons with AHD, including screening, diagnosis and treatment of TB and cryptococcal disease; prophylaxis for OIs, including TB preventive treatment (TPT), pre‐emptive antifungal treatment for those with cryptococcal antigenemia who do not have CM. For TB diagnosis, WHO recommends laboratory testing using GeneXpert® MTB/RIF, a nucleic acid‐based molecular test, and urine lateral flow lipoarabinomannan assay (LF‐LAM) antigen test for those with signs and symptoms of TB, and/or seriously ill, or with AHD [11]. Cryptococcal screening is recommended for PLHIV with CD4 <200 cells/mm^3^ using serum cryptococcal antigen (CrAg) [12].

With “Test and Treat,” there has been a shift from CD4 testing, including at baseline, to scale up of viral load testing for monitoring ART effectiveness. The reduction in CD4 testing has limited AHD identification with implications for CD4‐driven aspects of the WHO AHD package [10]. Therefore, we sought to develop an evidence‐based model to evaluate select components of the AHD package to determine the impact of different implementation scenarios on reducing mortality from TB and CM in high HIV prevalence countries.

In this analysis, we present a pair of models that link the presence or absence of baseline CD4 testing with selected components of the WHO AHD package of care for TB and cryptococcal disease. We applied these models to projections of HIV and TB disease mortality in the first year of ART for nine TB/HIV high‐burden countries. The implications of this analysis can inform country‐level discussions about the value of baseline CD4 testing in the prevention of mortality in the context of testing costs and requirements for key OIs.

## 2 METHODS

We developed two models to study the potential impact of forgoing baseline CD4 testing on mortality from TB and CM among patients with AHD in the first year of ART. Both models are based on national HIV estimates derived from the AIDS Impact Module (AIM) [13]. National AIM files, (which provide country‐level estimates of HIV prevalence, incidence and mortality) are publicly available and are updated and validated annually by the Ministry of Health of each country in a process coordinated by the Joint United Nations Programme on HIV/AIDS (UNAIDS), and this analysis uses AIM estimates of PLHIV by CD4 count and ART status from the 2021 files. The TB model is further based on TIME Estimates, a statistical model developed to project TB/HIV incidence and mortality using a regression approach applied to national HIV and TB estimates published by the WHO Global TB Programme and UNAIDS [14]. TB incidence estimates stratified by CD4 count and ART status among PLHIV were generated using the TIME model. Both AIM and TIME Estimates are implemented in the Spectrum suite of models developed by Avenir Health [15].

The TB and CM models were applied separately and independently. For each disease, we estimated the number of deaths in the first year of ART over 2019–2024, based on projections from AIM and TIME Estimates and case fatality ratios (CFRs) in the absence of AHD‐specific protocols. We then used these models to calculate the reduction in mortality with and without baseline CD4 testing. The mortality reduction depended on assumptions and estimates drawn from published literature about the availability of diagnostic tests, diagnostic sensitivity and specificity, and treatment coverage and efficacy [5, 11, 14, 16, 17, 18, 19] (see Table 2). The analysis was performed for nine of the 30 TB/HIV high‐burden countries identified by the Stop TB Partnership: Democratic Republic of Congo, Kenya, Lesotho, Mozambique, Nigeria, South Africa, Uganda, Zambia and Zimbabwe [20]. A one‐way sensitivity analysis was performed, where select model parameters were varied over a range of plausible values to gauge the impact on deaths averted by CD4 testing.

This analysis fell within routine programme monitoring and evaluation and did not involve contact with human subjects or personally identifiable information of human subjects. In addition, all aggregated programmatic, survey and census data were routinely collected. The analysis was approved by the CDC Center for Global Health as non‐human subjects research for public health programme activity.

### 2.1 TB model

**Figure 1:**
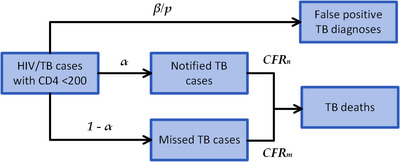
Tuberculosis (TB) model structure. Abbreviations: α, aggregate diagnostic sensitivity; β, aggregate diagnostic specificity; CFR_m_, case fatality ratio missed TB cases; CFR_n_, case fatality ratio notified TB cases; p, assumed TB prevalence among the screening population.

The TB model structure begins with the projected annual number of incident TB cases among new ART patients with CD4 <200 cells/mm^3^ at the onset of active TB disease, drawn from TIME Estimates (Figure 1, and Tables 1 and 2). TB cases are either notified or not notified with aggregate diagnostic sensitivity *α*, and CFRs are applied separately to notified and non‐notified cases. Finally, we calculate an estimate of the number of false‐positive TB diagnoses via an aggregate diagnostic specificity β and an assumed TB prevalence *p* among the screening population.

**Table 1:** Key parameter values for the tuberculosis (TB) model

Parameter	Definition	ART < 1 year	
**CFR_n_**	Case fatality ratio, notified TB cases [21]	6%	
**CFR_m_**	Case fatality ratio, missed TB cases [21]	62%	
**p**	Prevalence of TB in the screening population [11]	25%	

	**Calculated aggregate diagnostic sensitivity and specificity**	**With CD4**	**Without CD4**
**α**	Diagnostic sensitivity for TB, CD4 100–199 cells/mm^3^	88.1%	71.1%
**α**	Diagnostic sensitivity for TB, CD4 0–99 cells/mm^3^	88.1%	73.9%
**β**	Diagnostic specificity for TB, CD4 <200 cells/mm^3^	97.2%	94.5%

Note: The 2019 WHO TB LF‐LAM policy guidance summarizes several studies, with TB prevalence between 10% and 39% [11].

Abbreviations: ART, antiretroviral therapy; α, aggregate diagnostic sensitivity; β, aggregate diagnostic specificity; CFRm, case fatality ratio missed TB cases; CFRn, case fatality ratio notified TB cases; p, assumed TB prevalence among the screening population.

**Table 2:** Tuberculosis (TB) model parameters associated with diagnostic sensitivity, specificity and pathway mixture

	Not seriously ill		Seriously ill	
Empirical diagnostic yield by pathway ($\alpha_{i}$), CD4 <200 cells/mm^3^ [16]	With LF‐LAM	Without LF‐LAM	With LF‐LAM	Source
Clinical signs only	80.6%	39.0%	79.1%	Calculated [16]
Clinical signs & smear microscopy	82.9%	63.6%	82.7%	[16]
Clinical signs & smear microscopy & X‐ray	89.1%	72.9%	89.2%	[16]
Clinical signs & Xpert in sputum	87.6%	75.2%	87.8%	[16]
Clinical signs & Xpert in sputum & X‐ray	93.0%	82.9%	93.5%	[16]

**Specificity of screening and diagnostic tests (** $\beta_{s}$ **and** $\beta_{d}$)	**Value**	**Upper**	**Lower**	
Specificity of “any TB symptom,” for settings with high HIV prevalence	74%	53%	95%	[24]
Specificity X‐ray screening for “Any abnormality compatible with TB (active or inactive)”	75%	72%	79%	[24]
Specificity of conventional sputum smear microscopy	98%	93%	100%	[24]
Specificity of GeneXpert for active TB detection	99%	99%	99%	[22]
Specificity of LF‐LAM, CD4 <200 cells/mm^3^. Similar for lower CD4 categories	90%	78%	95%	[22]

**Calculated diagnostic specificity by pathway (** $\beta_{i}$ **), CD4 <200** cells/mm^3^	**With LF‐LAM**	**Without LF‐LAM**		
Clinical signs only	97.4%	74.0%		Calculated
Clinical signs & smear microscopy	96.9%	99.5%		Calculated
Clinical signs & smear microscopy & X‐ray	97.1%	99.5%		Calculated
Clinical signs & Xpert in sputum	97.2%	99.7%		Calculated
Clinical signs & Xpert in sputum & X‐ray	97.3%	99.8%		Calculated

**Other diagnostic parameters (assumed)**	**Value**			
Coverage of chest X‐ray for screening (vs. clinical signs)	75%			Assumed
Coverage of GeneXpert (vs. sputum smear microscopy)	60%			Assumed
Proportion who cannot generate sputum, or no sputum test available	20%			[25]
Proportion of HIV positive who are seriously ill, CD4 100–199 cells/mm^3^	10%			Assumed
Proportion of HIV positive who are seriously ill, CD4 0–99 cells/mm^3^	25%			Assumed

**Calculated diagnostic pathway mixture (** $w_{i}$)	**Coverage**			
Clinical signs only	20%			Calculated
Clinical signs & smear microscopy	8%			Calculated
Clinical signs & smear microscopy & X‐ray	24%			Calculated
Clinical signs & Xpert in sputum	12%			Calculated
Clinical signs & Xpert in sputum & X‐ray	36%			Calculated

Notes: The diagnostic yield for clinical signs only without LF‐LAM is computed assuming that in the clinical signs & LF‐LAM case, diagnosis follows a positive result from either clinical signs or LF‐LAM, and that the test results are independent. The addition of LF‐LAM reduces specificity for all pathways except “clinical signs only.” This follows from our assumption that a positive TB diagnosis follows from a positive result of any test, rather than all tests; the addition of any test with less than perfect specificity at the diagnostic stage will reduce $\beta_{d}$ and, therefore, overall pathway specificity. However, this effect is overwhelmed by the increase in specificity for the “clinical signs only” pathway such that aggregate specificity (Table 1) is still higher with CD4 and LF‐LAM.

Abbreviations: LF‐LAM, lateral flow urine lipoarabinomannan assay.

The aggregate diagnostic sensitivity and specificity are calculated based on a mixture of five diagnostic pathways representative of current TB diagnostic practice (Table 2). We assume a two‐stage diagnostic algorithm comprising five common two‐stage diagnostic pathways, with screening by clinical signs or X‐ray, followed by laboratory testing by either smear microscopy or GeneXpert, and LF‐LAM for patients with identified AHD. The influence of CD4 testing enters via eligibility for the LF‐LAM test. The pathway mixture is determined by assumed coverages of X‐ray (supplementing screening by clinical signs) and GeneXpert (replacing smear microscopy), giving a weight $w_{i}$ (with $\sum w_{i}=1$) for each diagnostic pathway sensitivity $\alpha_{i}$ and specificity $\beta_{i}$. The diagnostic sensitivity of each pathway $\alpha_{i}$ is given by empirical diagnostic yields measured in a clinical population with AHD [16]. The diagnostic specificity $\beta_{i}$ of each pathway is calculated from published estimates of individual test specificities [22, 23, 24], based on a two‐stage model of screening by either clinical signs and symptoms or X‐ray ($\beta_{s}$), followed by laboratory testing ($\beta_{d}$) by smear or Xpert, either alone or in parallel with LF‐LAM, using the formula $\beta_{i}=\beta_{s}+\left(1-\beta_{s}\right)\beta_{d}$. The aggregate sensitivity α and specificity β are computed as $\alpha =\sum w_{i}\alpha_{i}$ and $\beta =\sum w_{i}\beta_{i}$, where the sums are taken over the five diagnostic pathways.

The LF‐LAM test is used as an adjunct to sputum testing for patients with low CD4 counts and can aid in diagnosis for patients who cannot generate sputum [16]. The sensitivity and specificity of LF‐LAM depend on the extent of immunosuppression; diagnostic sensitivity is higher in patients with more severe immunosuppression [23]. The high specificity of LF‐LAM for patients with CD4 <200 cells/mm^3^ (range 78–95%), relative to clinical diagnosis alone, makes it useful in ruling out TB in patients with AHD, particularly if they cannot generate sputum [23]. At the time the model was designed, the WHO recommended LF‐LAM only if the patient was seriously ill (regardless of CD4), or had CD4 <100 cells/mm^3^ with TB signs and symptoms [2]. The WHO 2019 policy update has since expanded the recommendations to include inpatients with AHD irrespective of signs and symptoms of TB, or outpatients with CD4 <100 cells/mm^3^, irrespective of signs and symptoms of TB [11].

We assumed that LF‐LAM is universally available and used for WHO‐recommended eligible patients in addition to other diagnostic tools. Regardless of the presence or absence of CD4 testing, we assume that a proportion of patients present as severely ill and automatically qualify for LF‐LAM, and that an independent proportion cannot generate sputum and may be diagnosed only via clinical signs or LF‐LAM if identified as eligible. While the availability of CD4 testing has other benefits for the clinical management of AHD cases that are not captured here, the impact of CD4 testing in the TB model comes entirely through the increased use of LF‐LAM made possible by CD4 results.

### 2.2 CM model

**Figure 2:**
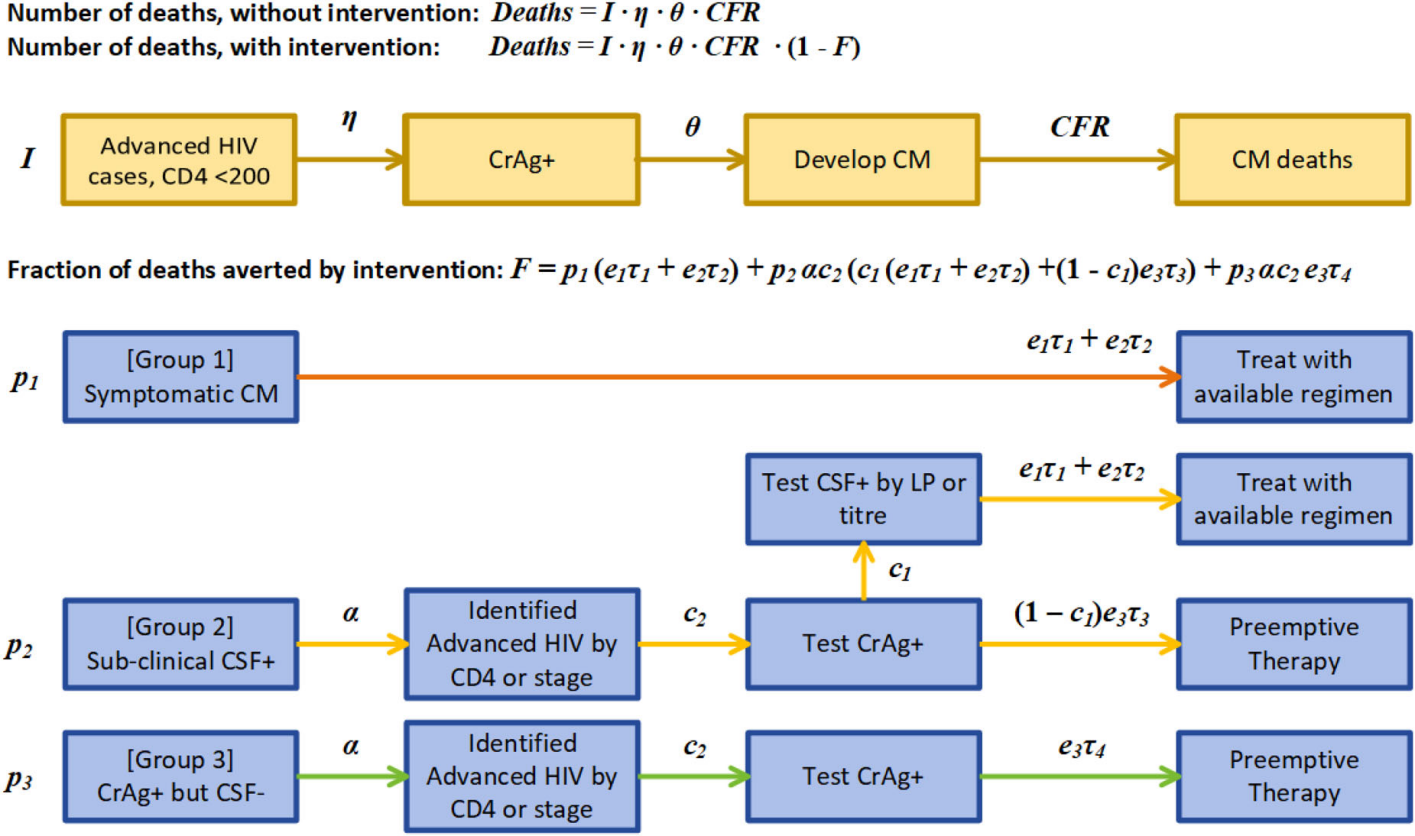
Cryptococcal meningitis (CM) model structure. The population is divided into three distinct groups: Group 1 with symptomatic CM at presentation, Group 2 with sub‐clinical CSF+ CM and Group 3 with CSF– CM. The model assumes two treatment regimens and a pre‐emptive therapy regimen, each characterized by a coverage $e_{i}$ and an efficacy $\tau_{i}$. Group 1 are assumed to be easily diagnosed and put on the available treatment regimen. Groups 2 and 3 must be first correctly identified as advanced HIV cases (α) in order to be screened for CM. If identified, Group 2 receive either a test for CSF‐positivity (*c*_1_) followed by the available treatment regimen, or else a test for cryptococcal antigenemia, if available (*c*_2_), followed by preventive therapy. If identified as advanced HIV, Group 3 receive a test for cryptococcal antigenemia, if available, followed by preventive therapy. See Table 3 for parameter values. Abbreviations: CrAg, cryptococcal antigen; CSF, cerebrospinal fluid.

The CM model begins with the projected number of PLHIV‐initiating ART with CD4 <200 cells/mm^3^, drawn from 2021 national AIM files in Spectrum (Figure 2 and Table 3). We then calculate the number of cryptococcal deaths that would be expected in the absence of any CM screening/treatment intervention, based on the prevalence η of cryptococcal antigenemia (CrAg+), the fraction θ of CrAg+ who go on to develop CM and a CFR for CM. Values for these parameters were drawn from expert opinion and published studies [5, 6, 17, 18, 19, 26]. Finally, we reduce these deaths based on the fraction *F* that are averted by intervention, where the value of *F* depends on whether AHD cases are identified by CD4 testing or clinical staging.

**Table 3:** Cryptococcal meningitis (CM) model parameters

		CD4 100–199	CD4 0–99	Source
*I*	Number of new HIV cases with CD4 < 200, from Spectrum/AIM	Varies by country	Varies by country	AIM
η	Prevalence of cryptococcal antigenemia among newly diagnosed HIV	2%	6.5%	[5]
θ *a*	Proportion of CrAg+, HIV positive on ART, who will develop CM without pre‐emptive therapy	70%	70%	[5]
CFR	Case fatality ratio for untreated CM	100%	100%	[5]

		**With CD4**	**Without CD4**	
α	Diagnostic sensitivity for advanced HIV	100%	60%	Assumed [17]
*e*	Fraction of deaths averted by intervention	52.5%	34.0%	Calculated
*p*_1_	Proportion of CrAg+ with symptomatic CM Group 2, sub‐clinical CrAg+	15%		[19]
*p*_2_	Proportion of CrAg+ with sub‐clinical CSF+ CM: Group 2, sub‐clinical CrAg+	48%		[19]
*p*_3_	Proportion of CrAg+ without CM (CSF‐negative): Group 3, CrAg+ and CSF– (p3 = 1 – p1 – p2)	37%		Calculated
*c*_1_	Coverage of test for CSF‐positivity by lumbar puncture for asymptomatic CrAg+	20%		Assumed
*c*_2_	Coverage of test for cryptococcal antigenemia	90%		Assumed
*e*_1_	Coverage of regimen 1, amphotericin‐based treatment	10%		Assumed
*e*_2_	Coverage of regimen 2, high‐dose fluconazole treatment (1–e1)	90%		Calculated
*E*_3_	Coverage of regimen 3, pre‐emptive therapy for CrAg+	90%		Assumed
τ_1_	Treatment efficacy of regimen 1	65%		[18]
τ_2_	Treatment efficacy of regimen 2	40%		[18]
τ_3_	Treatment efficacy of regimen 3	40%		Assumed
τ_4_	Treatment efficacy of regimen 3—CrAg+ but CSF–	100%		Assumed

Abbreviations: AIM, AIDS Impact Module; ART, antiretroviral therapy; CM, cryptococcal meningitis; CrAg, cryptococcal antigen; CSF, cerebrospinal fluid.

The fraction *F* of deaths averted by intervention is calculated by dividing CrAg+ AHD patients into three groups: those with symptomatic (we assume that multiple symptoms of CM would be detected in this group) CM at presentation (Group 1, *p*_1_), those with sub‐clinical CSF‐positive cryptococcal disease (Group 2, *p*_2_) and those with CSF‐negative cryptococcal antigenemia (CrAg+ but CSF–, Group 3, *p*_3_), such that $p_{1}+p_{2}+p_{3}=1$. The model assumes three treatment regimens, each associated with a coverage $e_{i}$, and four treatment efficacies $\tau_{i}$ of preventing death from CM. The first treatment is an amphotericin‐based regimen, while the second is a high‐dose fluconazole regimen, used when amphotericin is unavailable ($e_{2}=1-e_{1}$). We assume that in many settings, patients with CM receive fluconazole monotherapy, despite it no longer being a WHO‐recommended regimen, and that coverage of amphotericin‐based treatments is relatively low. The third treatment is fluconazole‐based preventive therapy for CrAg‐positive patients who are CSF‐negative (efficacy τ_3_), or who have sub‐clinical CM that is not detected by CSF CrAg testing (efficacy τ_4_). This third regimen is available with independent coverage *e*_3_.

We assume Group 1, those with symptomatic CM, are easily diagnosed and receive whichever of the two CM treatment regimens is available. Groups 2 and 3 must first be screened for AHD and correctly identified as such (*α*). If identified as AHD, Group 2 receive a test for cryptococcal antigenemia (coverage *c*_2_), followed a test for CSF‐positivity (coverage *c*_1_); a positive CSF test is followed by the available treatment regimen, while a negative CSF test is followed by pre‐emptive therapy. If identified as AHD, Group 3 receive a test for cryptococcal antigenemia followed by pre‐emptive therapy.

As with the TB model, the effect of CD4 testing on cryptococcal disease/CM mortality comes entirely through the identification of AHD, in this case for Groups 2 and 3. We assume that testing for both CrAg‐positivity and CSF‐positivity was indicated equally for all AHD cases, whether identified by clinical staging or CD4 testing. We assumed that CD4 testing would successfully identify all PLHIV with CD4 <200 cells/mm^3^ as having AHD. There are limitations of clinical staging for the identification of CD4 <200 cells/mm^3^; estimates of the sensitivity of clinical staging can vary but results from Munthali et al. showed that 60% of AHD cases (CD4 <200 cells/mm^3^) were identified by clinical staging [26], which we have adopted in our analysis.

## 3 RESULTS

**Figure 3:**
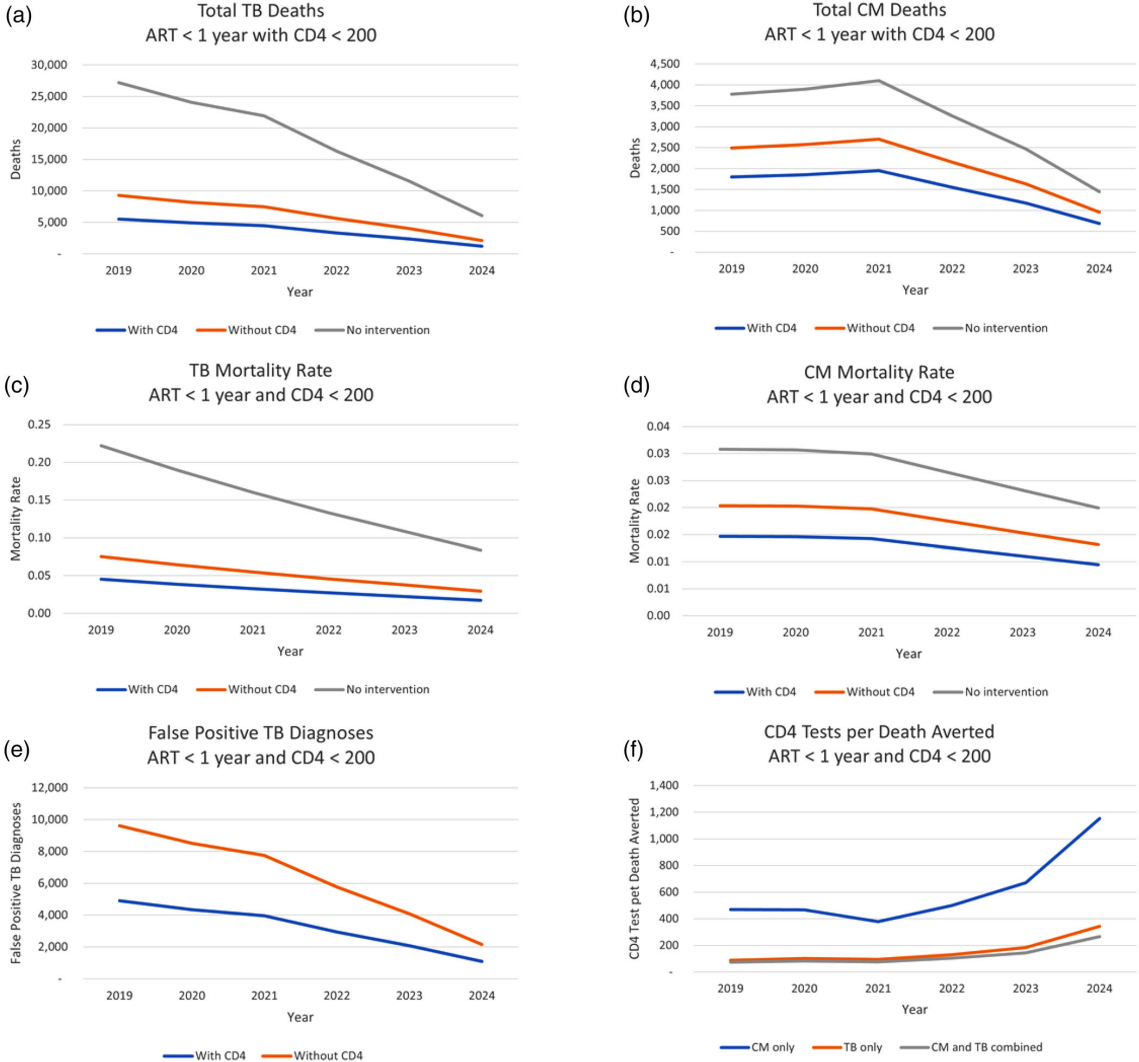
Selected results for South Africa, 2019–2024. Figures 3a and b show total deaths for the TB and CM models, respectively, for three scenarios: with CD4 testing, without CD4 testing and under no intervention (before application of the mortality reduction factor F). Figures 3c and d show the crude mortality rate from TB and CM for each scenario. Figure 3e shows the number of false‐positive TB diagnoses with and without CD4 testing. Figure 3f shows the number of CD4 tests that must be performed per death averted (deaths averted is deaths without CD4 testing minus deaths with CD4 testing) from CM only, TB only, and for CM and TB deaths combined. The overall decline in deaths and false‐positive diagnoses over 2019–2024 (a, b, e) is driven by a decline in the projected number of new ART initiations, as overall ART coverage expands, and patients initiate at higher CD4.

**Figure 4:**
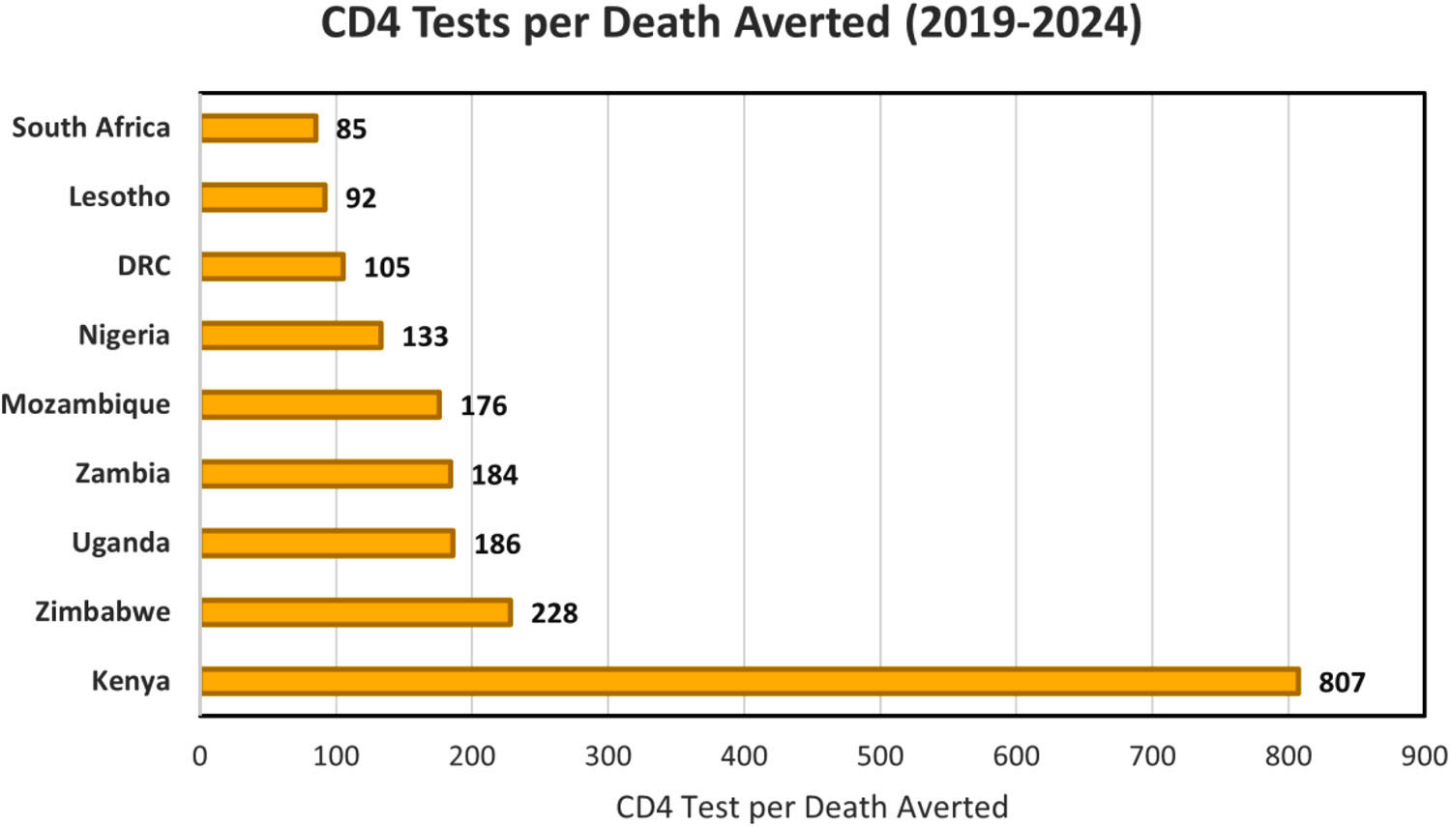
CD4 test per death averted from the TB and CM models combined, for nine countries. The number of CD4 tests per death averted from TB and CM combined, among ART <1 year with CD4 <200 cells/mm^3^, for the period 2019–2024. Countries vary by nearly an order of magnitude in the number of CD4 tests to avert deaths from TB and CM. Abbreviations: ART, antiretroviral therapy; CM, cryptococcal meningitis; TB, tuberculosis.

Selected results for South Africa are presented in detail (Figure 3 and Table 4); results for the other eight countries are qualitatively similar (Table 5 and Figure 4).

**Table 4:** Sensitivity analysis of changes in tuberculosis (TB) and cryptococcal meningitis (CM) deaths averted from the base value, South Africa

		Parameter value limit		Deaths averted^a^		Change in deaths averted^a^ (from base value)	
Parameter	Base value (%)	Lower (%)	Upper (%)	Lower	Upper	Lower	Upper
TB model							
Coverage of chest X‐ray for screening	75	50	80	15,265	14,770	412	–82
Coverage of GeneXpert	60	50	100	15,249	13,267	397	–1586
CM model							
α	60	45	73	4801	2357	1309	–1135
c_2_	90	10	100	388	3879	–3103	388
e_3_	90	50	100	2063	3848	–1428	357
e_1_	10	0	75	3475	3598	–16	106
c_1_	20	5	50	3459	3555	–32	64
t_1_	65	63	73	3490	3497	–1	5

Abbreviations: α, diagnostic sensitivity for advanced HIV disease without CD4 testing; c_2_, coverage of test for cryptococcal antigenemia; e_3_, coverage of regimen 3, pre‐emptive therapy for CrAg+; e_1_, coverage of regimen 1, amphotericin‐based treatment; c_1_, coverage of test for CSF‐positivity by lumbar puncture for asymptomatic CrAg+; t_1_, treatment efficacy of regimen 1.

^a^ Deaths averted for the TB model refers to TB deaths averted; deaths averted for the CM model refers to CM deaths averted.

**Table 5:** Extended results for all countries, 2019–2024

	South Africa	Kenya	Nigeria	Lesotho	Uganda	Mozambique	Zambia	DRC	Zimbabwe
CD4 tests per death averted	101	917	152	107	196	207	193	127	280
% of TB and CM deaths averted	37.3%	34.9%	33.2%	37.7%	36.9%	31.8%	37.3%	34.7%	31.2%
% of TB deaths averted	40.4%	41.6%	39.9%	41.2%	41.3%	40.6%	41.6%	40.8%	41.0%
% of CM deaths averted	27.9%	27.9%	27.9%	27.9%	27.9%	27.9%	27.9%	27.9%	27.9%
FP TB diagnoses averted	49.0%	49.0%	49.0%	49.0%	49.0%	49.0%	49.0%	49.0%	49.0%
TB deaths, with CD4	21,872	328	1414	773	1887	1651	2223	1283	530
TB deaths, without CD4	36,725	561	2354	1314	3215	2778	3810	2167	898
TB deaths averted	14,853	233	940	541	1328	1127	1587	884	368
CM deaths, with CD4	9004	393	2185	339	1135	4590	1264	1391	1957
CM deaths, without CD4	12,496	545	3033	470	1576	6370	1754	1931	2716
CM deaths averted	3491	152	847	131	440	1780	490	539	759
Combined deaths with CD4	30,877	721	3599	1112	3022	6242	3487	2674	2487
Combined deaths without CD4	49,221	1106	5386	1784	4791	9148	5564	4098	3614
Combined deaths averted	18,344	385	1788	672	1768	2907	2077	1423	1127

Abbreviations: CM, cryptococcal meningitis; FP, false‐positive; TB, tuberculosis.

### 3.1 TB model: South Africa

The presence of baseline CD4 testing enables LF‐LAM use in TB diagnosis for all new PLHIV with CD4 <200 cells/mm^3^, not just those who are seriously ill. The resulting increase in diagnostic sensitivity, especially for patients who cannot generate sputum, averts 40% of all TB deaths (14,853 deaths) that would occur over this 6‐year period without CD4/LF‐LAM (Figure 3a). The crude mortality rate in this group, that is the fraction of all HIV cases who would die from TB in their first year of ART, is reduced by a similar proportion from 5.3% to 3.2%. The average CFR due to TB is reduced from 21% to 13%.

### 3.2 CM model: South Africa

The use of CD4 to identify those in need of CrAg screening increases the number tested for cryptococcal antigenemia and sub‐clinical CSF+ cryptococcal disease and averts 27.9% of deaths (3491 deaths) relative to CrAg screening based on clinical staging alone (Figure 3b). The crude mortality rate in this group, that is the fraction of all HIV cases who would die from cryptococcal disease in their first year of treatment, is reduced by a similar proportion from 1.8% to 1.3% (Figure 3d). The CFR for CM is reduced from 66% to 48%.

### 3.3 TB and CM models combined: South Africa

Both models naturally account for the overlap between TB and CM, since they start from projections of HIV and TB cases and apply CFRs that were measured in the presence of both diseases. The presence of CD4 testing to identify AHD cases averted 37% of all deaths from TB and CM combined (18,344 deaths), compared to clinical staging alone (Table 5). Figure 3f shows the number of CD4 tests performed per death averted from TB only, from CM only, and from both diseases combined. A sensitivity analysis modifying select parameters in the TB and CM models notes changes in deaths averted from the base value based on their respective upper and lower parameter values (Table 4; see Tables S1–S8 for analysis of other countries).

### 3.4 Variation in model results by country: nine countries

The analysis described above for South Africa was repeated for eight additional high TB/HIV burden countries using the same model parameters described in Tables 1, 2, 3, but with country‐specific projections of TB and HIV burden. The results were qualitatively similar in most respects but varied over an order of magnitude in the number of CD4 tests required per death averted from TB and CM combined (Figure 4 and Table 5). The fraction of all TB and CM deaths averted utilizing CD4 testing varied slightly—between 31% and 38%. The number of TB and CM deaths averted utilizing CD4 testing ranged from 385 deaths for Kenya to 18,344 deaths for South Africa (Table 5).

The variation in model results—in particular, CD4 tests per death averted—warrants some commentary. As the CM and TB model parameters are the same for all countries, variation in the model results is due to variation in the inputs from the AIM and TIME estimates. In particular, the number and prevalence of PLHIV with AHD‐initiating ART vary across countries, as well as TB incidence among that group. Kenya appears to be an outlier for CD4 tests per death averted because of the lower TB incidence (from TIME estimates) among PLHIV with AHD‐initiating ART in Kenya relative to other countries.

## 4 DISCUSSION

CD4 testing allows the identification of the highest risk PLHIV who may benefit from evidence‐based interventions that reduce mortality from TB and CM. Our results show that CD4 testing, followed by appropriate OI screening prophylaxis, and treatment can potentially avert between 31% and 38% of deaths from TB and CM among PLHIV with AHD across a range of epidemiologic contexts in SSA. These findings are critically important given the continued high rate of AHD and underscore the importance of baseline CD4 testing on reducing preventable deaths due to TB and CM among PLHIV.

Our results show that CD4 testing followed by TB screening, prophylaxis and treatment can potentially avert approximately 40% of TB deaths. The impact of CD4 in averting deaths from TB was seen primarily in identifying PLHIV with AHD for LF‐LAM testing. Even though Xpert MTB/RIF remains the primary diagnostic test for all PLHIV undergoing evaluation for TB disease, LF‐LAM can aid in TB diagnosis for persons who are seriously ill or have AHD. The only LF‐LAM assay currently commercially available (Alere Determine TB LAM Ag) is a point‐of‐care (POC) test recommended by WHO since 2015 [11]. Data from hospitalized patients from four southern African countries found that LF‐LAM use as an adjunct to Xpert reduced mortality [27]. Despite this, LF‐LAM uptake has been limited for multiple reasons. A recent survey of 31 high TB/HIV burden countries found that fewer than half of them had policies in place for LF‐LAM use and only 21% were currently using LF‐LAM for diagnosis, in part due to confusion about whether TB programmes or HIV programmes should be responsible for implementation [28]. In addition, reduced funding for CD4 testing may have limited the uptake of this assay [10]. A more sensitive LAM assay, Fujifilm SILVAMP TB LAM (FujiLAM), has been developed and may increase LAM use [29]. Our data provide compelling information to help countries quantify the anticipated impact of CD4 testing and LF‐LAM use, in addition to other TB diagnostics, to save lives among PLHIV.

This analysis found that CD4 testing could potentially avert approximately 28% of deaths from CM, which is estimated to cause over 112,000 deaths annually [30]. Despite WHO recommendations for CrAg testing which are CD4‐based, CrAg screening in SSA is sub‐optimal and reductions in CD4 testing in the “Test and Treat” era have limited CrAg screening in certain settings [10]. It is likely that the efficacy of CD4‐directed CrAg screening and pre‐emptive antifungal treatment of CrAg‐positive patients will increase in the coming years; new regimens for pre‐emptive antifungal treatment are being evaluated in clinical trials which may improve outcomes in those with cryptococcal antigenemia [31]. New rapid diagnostics are also under evaluation which may aid in the detection of sub‐clinical CM via rapid detection of high CrAg titres in the blood of CrAg‐positive patients, thereby increasing the number of patients who are placed on appropriate CM therapy (even if lumbar puncture and CSF testing is not available) [19, 32]. These advances in diagnosis and treatment could lead to a more pronounced reduction in cryptococcal mortality in CD4‐based CrAg screening programmes in the future. In addition, access to gold‐standard CM treatment has improved recently, which may improve CM outcomes.

The analysis has several limitations. First, we do not account for uncertainties in the projected course of HIV or TB epidemiology, derived from the AIM and TIME Estimates models, the proportion of AHD detected by clinical staging, or other components of the AHD package known to reduce mortality like TPT or cotrimoxazole. Second, the benefits of CD4 testing in this analysis arise through increased use of LF‐LAM for TB diagnosis and improved identification of sub‐clinical CM and cryptococcal antigenemia; we do not account for other clinical benefits of CD4 testing and the lack of LF‐LAM use in routine clinical settings. Third, most model parameter values—although not HIV and TB incidence—are held constant over the 6‐year period. There is a good reason to expect the TB diagnostic algorithm to become more sensitive and specific as the utilization of TB diagnostic tests increases, which could diminish the marginal value of baseline CD4 testing. In addition, the expanded eligibility criteria for LF‐LAM testing do not include CD4. Fourth, the assumed benefits in our model may overestimate the benefits in routine utilization of baseline CD4, which is subject to implementation challenges and may lead to delayed results for CD4 and thus LF‐LAM or CrAg testing. Fifth, since the AIM model does not capture CD4 testing indicated for those returning to care, our model may underestimate the deaths averted. Sixth, we conservatively assume comprehensive CrAg testing for patients with clinical stage 3 or 4 illness, absent a CD4 result; some countries recommend this, but many countries do not yet implement this approach. Finally, since we performed limited sensitivity analyses, future modelling work should include multivariable sensitivity analyses to inform researchers and implementers about the robustness of the assumptions in these models.

## 5 CONCLUSIONS

This analysis provides evidence that baseline CD4 testing for newly diagnosed PLHIV, in addition to OI screening and prophylaxis/treatment, could avert deaths from the two most common and deadly OIs in ART‐naive patients. Rapid HIV diagnosis, timely linkage to ART and continuity of HIV treatment are essential to prevent AHD. The US President's Emergency Plan for AIDS Relief (PEPFAR) recommends CD4 testing for the identification of AHD in specific contexts (at the initiation of ART for PLHIV over 5 years, upon re‐initiation of care for those out of care for more than a year and for individuals with virologic failure) [33]. A new semi‐quantitative POC lateral flow assay, VISITECT CD4 advanced disease test which differentiates CD4 <200 or ≥200 cells/mm^3^ to rapidly identify AHD, has been WHO prequalified [34]. Optimization of current conventional CD4 platforms and targeted placement of this new CD4 test may improve access to CD4 and identification of AHD. However, national programmes will need to weigh the cost of increasing CD4 access against other HIV‐related priorities and allocate resources accordingly. Overall, our results support retaining baseline CD4 testing to strengthen the identification of AHD and reduce morbidity and mortality from TB and CM.

## COMPETING INTERESTS

The authors have no competing interests.

## AUTHORS’ CONTRIBUTIONS

IKO and HP: conception and design, study management, data collection and manuscript writing with input from CC, MH, AJ, HLK, EA, LJP, CP, GG, TC, MD, RB, RWS and NSS. CC and MH: technical advice for statistical aspects, data management, data analysis and manuscript writing. MH and AJ: conception and design. RWS and NSS: conception, design and critical revision of the manuscript. IKO and HP made revisions with input from co‐authors. All authors read and approved the final manuscript.

## FUNDING

This project has been supported by the President's Emergency Plan for AIDS Relief (PEPFAR) through the Centers for Disease Control and Prevention (CDC) under a cooperative agreement, CoAg # U2GGH000994.

## DISCLAIMER

The findings and conclusions in this manuscript are those of the authors and do not necessarily represent the official position of the funding agencies. The funders had no role in study design, data collection and analysis, decision to publish, or preparation of the manuscript.

## Supporting information


**Supplemental Table 1**: Changes in TB and CM deaths averted from base value, Kenya.
**Supplemental Table 2**: Changes in TB and CM deaths averted from base value, Nigeria.
**Supplemental Table 3**: Changes in TB and CM deaths averted from base value, Lesotho.
**Supplemental Table 4**: Changes in TB and CM deaths averted from base value, Uganda.
**Supplemental Table 5**: Changes in TB and CM deaths averted from base value, Mozambique.
**Supplemental Table 6**: Changes in TB and CM deaths averted from base value, Zambia.
**Supplemental Table 7**: Changes in TB and CM deaths averted from base value, Democratic Republic of Congo (DRC).
**Supplemental Table 8**: Changes in TB and CM deaths averted from base value, Zimbabwe.

## Data Availability

The data that support the findings of this study are available from the corresponding author upon reasonable request.
