# Supplementary material for: Modelling the impact of CD4 testing on mortality from TB and cryptococcal meningitis among patients with advanced HIV disease in nine countries
Source: J Int AIDS Soc. 2023 Mar 7;26(3):e26070. doi: 10.1002/jia2.26070 (PMC9989935; doi:10.1002/jia2.26070)
Supplement: Supplementary file 1 — Supplemental Table 1: Changes in TB and CM deaths averted from base value, Kenya. Supplemental Table 2: Changes in TB and CM deaths averted from base value, Nigeria. Supplemental Table 3: Changes in TB and CM deaths averted from base value, Lesotho. Supplemental Table 4: Changes in TB and CM deaths averted from base value, Uganda. Supplemental Table 5: Changes in TB and CM deaths averted from base value, Mozambique. Supplemental Table 6: Changes in TB and CM deaths averted from base value, Zambia. Supplemental Table 7: Changes in TB and CM deaths averted from base value, Democratic Republic of Congo (DRC). Supplemental Table 8: Changes in TB and CM deaths averted from base value, Zimbabwe. [file JIA2-26-e26070-s001.docx]

**Supplemental Table 1: Changes in TB and CM deaths averted from base value, Kenya**

| Kenya | | | | | | | |
| --- | --- | --- | --- | --- | --- | --- | --- |
|  | | Parameter Value Limit | | Deaths Averted* | | Change in Deaths Averted * (from Base Value) | |
| Parameter | Base Value (%) | Lower (%) | Upper (%) | Lower | Upper | Lower | Upper |
| TB Model | | | | | | | |
| Coverage of chest x-ray for screening | 75 | 50 | 80 | 239 | 231 | 6 | -2 |
| Coverage of GeneXpert | 60 | 50 | 100 | 239 | 208 | 6 | -25 |
| CM Model | | | | | | | |
| α | 60 | 45 | 73 | 210 | 103 | 58 | -49 |
| c_2_ | 90 | 10 | 100 | 17 | 169 | -135 | 17 |
| e_3_ | 90 | 50 | 100 | 90 | 168 | -62 | 16 |
| e_1_ | 10 | 0 | 75 | 152 | 157 | 0 | 5 |
| c_1_ | 20 | 5 | 50 | 151 | 155 | -1 | 3 |
| t_1_ | 65 | 63 | 73 | 152 | 153 | 0 | 1 |
| *Deaths averted for the TB model refers to TB deaths averted; deaths averted for the CM model refers to CM deaths averted.  Abbreviations: AIM: AIDS Impact Module, ART: antiretroviral therapy, CM: cryptococcal meningitis, CrAg: Cryptococcal Antigen, α: Diagnostic sensitivity for advanced HIV disease without CD4 testing, c_2_: Coverage of test for cryptococcal antigenemia, e_3_: Coverage of regimen 3, pre-emptive therapy for CrAg+, e_1_: Coverage of regimen 1, Amphotericin-based treatment, c_1_: Coverage of test for CSF-positivity by lumbar puncture for asymptomatic CrAg+, t_1_: Treatment efficacy of regimen 1. | | | | | | | |

**Supplemental Table 2: Changes in TB and CM deaths averted from base value, Nigeria**

| Nigeria | | | | | | | |
| --- | --- | --- | --- | --- | --- | --- | --- |
|  | | Parameter Value Limit | | Deaths Averted* | | Change in Deaths Averted * (from Base Value) | |
| Parameter | Base Value (%) | Lower (%) | Upper (%) | Lower | Upper | Lower | Upper |
| TB Model | | | | | | | |
| Coverage of chest x-ray for screening | 75 | 50 | 80 | 966 | 935 | 26 | -5 |
| Coverage of GeneXpert | 60 | 50 | 100 | 965 | 840 | 25 | -100 |
| CM Model | | | | | | | |
| α | 60 | 45 | 73 | 1165 | 572 | 318 | -275 |
| c_2_ | 90 | 10 | 100 | 94 | 941 | -753 | 94 |
| e_3_ | 90 | 50 | 100 | 501 | 934 | -346 | 87 |
| e_1_ | 10 | 0 | 75 | 843 | 873 | -4 | 26 |
| c_1_ | 20 | 5 | 50 | 840 | 863 | -7 | 16 |
| t_1_ | 65 | 63 | 73 | 847 | 849 | 0 | 2 |
| *Deaths averted for the TB model refers to TB deaths averted; deaths averted for the CM model refers to CM deaths averted. | | | | | | | |

**Supplemental Table 3: Changes in TB and CM deaths averted from base value, Lesotho**

| Lesotho | | | | | | | |
| --- | --- | --- | --- | --- | --- | --- | --- |
|  | | Parameter Value Limit | | Deaths Averted* | | Change in Deaths Averted * (from Base Value) | |
| Parameter | Base Value (%) | Lower (%) | Upper (%) | Lower | Upper | Lower | Upper |
| TB Model | | | | | | | |
| Coverage of chest x-ray for screening | 75 | 50 | 80 | 556 | 538 | 15 | -3 |
| Coverage of GeneXpert | 60 | 50 | 100 | 555 | 483 | 14 | -58 |
| CM Model | | | | | | | |
| α | 60 | 45 | 73 | 181 | 89 | 50 | -42 |
| c_2_ | 90 | 10 | 100 | 15 | 146 | -116 | 15 |
| e_3_ | 90 | 50 | 100 | 78 | 145 | -53 | 14 |
| e_1_ | 10 | 0 | 75 | 131 | 135 | 0 | 4 |
| c_1_ | 20 | 5 | 50 | 130 | 134 | -1 | 3 |
| t_1_ | 65 | 63 | 73 | 131 | 132 | 0 | 1 |
| *Deaths averted for the TB model refers to TB deaths averted; deaths averted for the CM model refers to CM deaths averted. | | | | | | | |

**Supplemental Table 4: Changes in TB and CM deaths averted from base value, Uganda**

| Uganda | | | | | | | |
| --- | --- | --- | --- | --- | --- | --- | --- |
|  | | Parameter Value Limit | | Deaths Averted* | | Change in Deaths Averted * (from Base Value) | |
| Parameter | Base Value (%) | Lower (%) | Upper (%) | Lower | Upper | Lower | Upper |
| TB Model | | | | | | | |
| Coverage of chest x-ray for screening | 75 | 50 | 80 | 1365 | 1321 | 37 | -7 |
| Coverage of GeneXpert | 60 | 50 | 100 | 1363 | 1186 | 35 | -142 |
| CM Model | | | | | | | |
| α | 60 | 45 | 73 | 181 | 89 | 605 | 297 |
| c_2_ | 90 | 10 | 100 | 15 | 146 | 49 | 489 |
| e_3_ | 90 | 50 | 100 | 78 | 145 | 260 | 485 |
| e_1_ | 10 | 0 | 75 | 131 | 135 | 438 | 454 |
| c_1_ | 20 | 5 | 50 | 130 | 134 | 436 | 448 |
| t_1_ | 65 | 63 | 73 | 131 | 132 | 440 | 441 |
| *Deaths averted for the TB model refers to TB deaths averted; deaths averted for the CM model refers to CM deaths averted. | | | | | | | |

**Supplemental Table 5: Changes in TB and CM deaths averted from base value, Mozambique**

| Mozambique | | | | | | | |
| --- | --- | --- | --- | --- | --- | --- | --- |
|  | | Parameter Value Limit | | Deaths Averted* | | Change in Deaths Averted * (from Base Value) | |
| Parameter | Base Value (%) | Lower (%) | Upper (%) | Lower | Upper | Lower | Upper |
| TB Model | | | | | | | |
| Coverage of chest x-ray for screening | 75 | 50 | 80 | 1158 | 1121 | 31 | -6 |
| Coverage of GeneXpert | 60 | 50 | 100 | 1157 | 1007 | 30 | -120 |
| CM Model | | | | | | | |
| α | 60 | 45 | 73 | 2447 | 1201 | 667 | -579 |
| c_2_ | 90 | 10 | 100 | 198 | 1978 | -1582 | 198 |
| e_3_ | 90 | 50 | 100 | 1052 | 1962 | -728 | 182 |
| e_1_ | 10 | 0 | 75 | 1772 | 1834 | -8 | 54 |
| c_1_ | 20 | 5 | 50 | 1764 | 1812 | -16 | 32 |
| t_1_ | 65 | 63 | 73 | 1779 | 1783 | -1 | 3 |
| *Deaths averted for the TB model refers to TB deaths averted; deaths averted for the CM model refers to CM deaths averted. | | | | | | | |

**Supplemental Table 6: Changes in TB and CM deaths averted from base value, Zambia**

| Zambia | | | | | | | |
| --- | --- | --- | --- | --- | --- | --- | --- |
|  | | Parameter Value Limit | | Deaths Averted* | | Change in Deaths Averted * (from Base Value) | |
| Parameter | Base Value (%) | Lower (%) | Upper (%) | Lower | Upper | Lower | Upper |
| TB Model | | | | | | | |
| Coverage of chest x-ray for screening | 75 | 50 | 80 | 1631 | 1578 | 44 | -9 |
| Coverage of GeneXpert | 60 | 50 | 100 | 1629 | 1417 | 42 | -170 |
| CM Model | | | | | | | |
| α | 60 | 45 | 73 | 674 | 331 | 184 | -159 |
| c_2_ | 90 | 10 | 100 | 54 | 545 | -436 | 55 |
| e_3_ | 90 | 50 | 100 | 290 | 540 | -200 | 50 |
| e_1_ | 10 | 0 | 75 | 488 | 505 | -2 | 15 |
| c_1_ | 20 | 5 | 50 | 486 | 499 | -4 | 9 |
| t_1_ | 65 | 63 | 73 | 490 | 491 | 0 | 1 |
| *Deaths averted for the TB model refers to TB deaths averted; deaths averted for the CM model refers to CM deaths averted. | | | | | | | |

**Supplemental Table 7: Changes in TB and CM deaths averted from base value, Democratic Republic of Congo (DRC)**

| DRC | | | | | | | |
| --- | --- | --- | --- | --- | --- | --- | --- |
|  | | Parameter Value Limit | | Deaths Averted* | | Change in Deaths Averted * (from Base Value) | |
| Parameter | Base Value (%) | Lower (%) | Upper (%) | Lower | Upper | Lower | Upper |
| TB Model | | | | | | | |
| Coverage of chest x-ray for screening | 75 | 50 | 80 | 908 | 879 | 24 | -5 |
| Coverage of GeneXpert | 60 | 50 | 100 | 908 | 790 | 24 | -94 |
| CM Model | | | | | | | |
| α | 60 | 45 | 73 | 742 | 364 | 203 | -175 |
| c_2_ | 90 | 10 | 100 | 60 | 599 | -479 | 60 |
| e_3_ | 90 | 50 | 100 | 319 | 595 | -220 | 56 |
| e_1_ | 10 | 0 | 75 | 537 | 556 | -2 | 17 |
| c_1_ | 20 | 5 | 50 | 534 | 549 | -5 | 10 |
| t_1_ | 65 | 63 | 73 | 539 | 540 | 0 | 1 |
| *Deaths averted for the TB model refers to TB deaths averted; deaths averted for the CM model refers to CM deaths averted. | | | | | | | |

**Supplemental Table 8: Changes in TB and CM deaths averted from base value, Zimbabwe**

| Zimbabwe | | | | | | | |
| --- | --- | --- | --- | --- | --- | --- | --- |
|  | | Parameter Value Limit | | Deaths Averted* | | Change in Deaths Averted * (from Base Value) | |
| Parameter | Base Value (%) | Lower (%) | Upper (%) | Lower | Upper | Lower | Upper |
| TB Model | | | | | | | |
| Coverage of chest x-ray for screening | 75 | 50 | 80 | 379 | 366 | 11 | -2 |
| Coverage of GeneXpert | 60 | 50 | 100 | 378 | 329 | 10 | -39 |
| CM Model | | | | | | | |
| α | 60 | 45 | 73 | 1043 | 512 | 284 | -247 |
| c_2_ | 90 | 10 | 100 | 84 | 843 | -675 | 84 |
| e_3_ | 90 | 50 | 100 | 448 | 836 | -311 | 77 |
| e_1_ | 10 | 0 | 75 | 755 | 782 | -4 | 23 |
| c_1_ | 20 | 5 | 50 | 752 | 773 | -7 | 14 |
| t_1_ | 65 | 63 | 73 | 759 | 760 | 0 | 1 |
| *Deaths averted for the TB model refers to TB deaths averted; deaths averted for the CM model refers to CM deaths averted. | | | | | | | |
